# A multiscale modelling approach for *Haematococcus pluvialis* cultivation under different environmental conditions

**DOI:** 10.1016/j.btre.2022.e00771

**Published:** 2022-10-19

**Authors:** Alessandro Usai, Jon K. Pittman, Constantinos Theodoropoulos

**Affiliations:** a Department of Chemical Engineering, University of Manchester, M13 9PL, UK; b Biochemical and Bioprocess Engineering Group, University of Manchester, M13 9PL, UK; c Department of Earth and Environmental Sciences, University of Manchester, M13 9PL, UK

**Keywords:** Microalgae, Batch cultivation, Cell lysis, Cell fission, Population balance, Nutrient limitation

## Abstract

*Haematococcus pluvialis* can produce significant amounts of industrially important compounds belonging to lipids and starch classes, including various specific pigments such as β-carotene, lutein and astaxanthin, as well as lipids, carbohydrates and proteins. Their production can vary depending on environmental stress conditions like nutrient starvation. However, stress conditions lead also to undesired phenomena such as cell lysis, which is likely to be related to products loss. The microorganism develops towards smaller single cell volumes during the growth process, and eventually, more likely towards lysis when fission (i.e. cell division) slows down. The lysis process takes place simultaneously with nutrient depletion, so both growth and lysis are linked to the change of environmental conditions. In this work, we develop a novel multiscale segregated-structured model based on Population Balance Equations (PBEs) to describe the photoautotrophic growth of *H.pluvialis*, in particular cell growth, and lysis, making possible the description of the relationship between cell volume/transition, cell loss, and metabolic product availability. Cell volume is the internal coordinate of the population balance model, and its link with intrinsic concentrations is also presented. The model parameters are fitted against experimental data, extensive sensitivity analysis is performed and the model predictive capabilities are tested in terms of cell density distributions, as well as 0th and 1st order moments.

## Highlights

• We develop a novel multiscale model for microalgal photoautotrophic growth.
• The model is segregated-structured type based on Population Balance Equations.
• We combine the model with cultivation experiments of Haematococcus pluvialis.
• We successfully predict cell number, average volume and density distribution dynamics.
• Model can accurately describe the nutrient depletion phase including cell lysis.

## Nomenclature

${C_{{\text{NO}}_{3}^{-}}}^{\text{Ext}}$: Extracellular nitrates concentration, g L^−1^
${C_{{\text{NO}}_{3}^{-}}}^{\text{Int}}$: Intracellular nitrates concentration, g L^−1^
${\dot{C}}_{{\text{NO}}_{3}^{-}}$: Nitrates transport rate, g h^−1^
$f_{V}$: Gaussian shape function, µm^−3^
$f_{{C_{{\text{NO}}_{3}^{-}}}^{\text{Int}}}$: Intracellular nitrates growth rate factor
$f_{I_{\text{Ave}},G}$: Average light growth rate factor
$f_{{C_{{\text{NO}}_{3}^{-}}}^{\text{Int}},L}$: Intracellular nitrates growth rate factor for low affinity kinetics
$f_{{C_{{\text{NO}}_{3}^{-}}}^{\text{Int}},H}$: Intracellular nitrates growth rate factor for high affinity kinetics
$f_{c_{{\text{NO}}_{3}^{-,0}}^{\text{Med}}}$: Initial nitrates fission rate factor
$f_{I_{0}}$: Incident light fission rate factor
$f_{{C_{{\text{NO}}_{3}^{-}}}^{\text{Ext}},V_{c}}$: Extracellular nitrates critical volume factor
$f_{{C_{{\text{NO}}_{3}^{-}}}^{\text{Int}},V_{c}}$: Intracellular nitrates critical volume factor
$hf_{1}$: Flex point initial nitrates fission inhibition, g L^−1^
$hf_{2}$: Flex point incident light fission inhibition, µmol m^−2^s^−1^
$h_{\text{min}}$: Minimum fission factor for incident light
$hs_{1}$: Shape factor initial nitrates fission inhibition, g L ^−^ ^1^
$hs_{2}$: Shape factor incident light fission inhibition, µmol m ^−^ ^2^s^−1^
$I_{0}$: Incident light intensity, µmol m^−2^s^−1^
$I_{\text{ave}}$: Average light density, µmol m^−2^s^−1^
$K_{{C_{{\text{NO}}_{3}^{-}}}^{\text{Ext}},V_{c}}$: Saturation constant external nitrates critical volume, g L^−1^
$K_{{C_{{\text{NO}}_{3}^{-}}}^{\text{Ext}},U}$: Saturation constant nitrates uptake, g L^−1^
$K_{{C_{{\text{NO}}_{3}^{-}}}^{\text{Int}},V_{c}}$: Saturation constant internal nitrates critical volume, g *L*^−1^
$K_{{C_{{\text{NO}}_{3}^{-}}}^{\text{Int}},C}$: Saturation constant nitrates consumption, g L^−1^
$K_{{C_{{\text{NO}}_{3}^{-}}}^{\text{Int}},C:I}$: Inhibition constant nitrates consumption, g L^−1^
$K_{{C_{{\text{NO}}_{3}^{-}}}^{\text{Int}},H}$: Saturation constant nitrates high-affinity growth and fission, g L^−1^
$K_{{C_{{\text{NO}}_{3}^{-}}}^{\text{Int}},H:I}$: Inhibition constant nitrates high-affinity growth and fission, g L^−1^
$K_{{C_{{\text{NO}}_{3}^{-}}}^{\text{Int}},L:I}$: Inhibition constant nitrates low-affinity growth and fission, g L^−1^
$K_{{C_{{\text{NO}}_{3}^{-}}}^{\text{Int}},\text{LG}}$: Saturation constant nitrates low-affinity growth and fission, g L^−1^
$K_{I_{\text{Ave},c}}$: Saturation constant average light consumption, µmol m^−2^s^−1^
$K_{I_{\text{Ave}},c:I}$: Inhibition constant average light consumption, µmol m^−2^s^−1^
$K_{I_{\text{Ave}}}$: Saturation constant average light growth and fission, µmol m^−2^s^−1^
$K_{I_{\text{Ave}}:I}$: Inhibition constant average light growth and fission, µmol m^−2^s^−1^
$k_{\text{Lys},0}$: Pre-exponential factor cell lysis, h^−1^
$k_{\text{Lys},S}$: Exponential factor cell lysis, h µm^−3^
$n_{c}$: Shape factor nitrates consumption
$n_{H}$: Shape factor nitrates high-affinity growth and fission
$n_{I}$: Shape factor average light growth and fission
$n_{\text{Ic}}$: Shape factor average light consumption
$n_{L}$: Shape factor nitrate low-affinity growth and fission
$p_{i}$: Partitioning continuous distribution function, µm^−3^
$P_{i}$: Normalised partitioning continuous distribution function, µm^−3^
$r_{f}$: Specific transition rate, µm^3^ h^−1^
$r_{G,{\text{NO}}_{3}^{-}}$: Nitrates consumption rate, g h^−1^ L^−1^
$r_{V}$: Cell volume growth rate, µm^3^ h^−1^
$v_{c,\text{max}}$: Maximum critical volume, µm^3^
$v_{c}$: Critical fission volume, µm^3^
$V_{\text{Cells}}^{T}$: Total intracellular cell volume, L
$V_{\text{Med}}^{T}$: Total extracellular media volume, L
$V_{R}^{T}$: Total reactor volume, L
$x_{A}$: Affinity factor nitrate growth and fission
$y_{A}$: Affinity factor critical volume
v: Cell volume, µm^3^
z: Vessel depth, m

**Greek letters**
$\alpha_{2}$: Hill-Ng Distribution 1st parameter, 2 Daughters
$\alpha_{4}$: Hill-Ng Distribution 1st parameter, 4 Daughters
β: Light attenuation coefficient, L g^−1^ m^−1^
$\Gamma^{f}$: Transition rate, h^−1^
$\gamma^{f}$: Gamma function, µm^−3^
$\delta_{2}$: Hill-Ng Distribution 2nd parameter, 2 Daughters
$\delta_{4}$: Hill-Ng Distribution 2nd parameter, 4 Daughters
$\Theta_{2}$: Probability of two daughter cells birth per mitotic event
$\mu_{\text{max}}$: Maximum specific growth rate, µm h^−1^
$\mu_{0}$: Mean initial value distribution, µm^3^
$\rho_{C,\text{Max}}$: Maximum rate of nitrates consumption, g h^−1^ L^−1^
$\rho_{\text{Cells}}$: Density of the cell, g dm^−3^
$\rho_{U,\text{Max}}$: Maximum rate nitrates uptake, g h^−1^ L^−1^
$\sigma_{0}$: Standard deviation initial distribution, µm^3^
$\sigma_{c}$: Standard deviation critical distribution, µm^3^
$\Psi_{V}$: Density distribution function, µm^−3^mL^−1^

## 1 Introduction

Microalgae as biofactories of the future are currently facing an increasing interest by researchers and industrial biotechnologists, due to their potential to produce a wide range of high added-value products in a biorefinery context, such as biofuels, fertilizers, antioxidants, nutraceuticals as well as anti-inflammatory, and antimicrobial substances [1,2]. Microalgal biotechnology is a viable candidate to help tackle the effects of fossil fuels depletion by producing third-generation biofuels and treating wastewater through the consumption of NH_4_^+^, NO_3_^−^, and PO_4_^3−^. Moreover, microalgae metabolites can be an essential source of bio-derived products used in many industrial applications [3]. Photoautotrophic microalgae can also fixate carbon dioxide (CO_2_) by using it as a carbon source to produce cellular material macromolecules and metabolites. Hence, their cultivation is a promising tool for CO_2_ sequestration [4] and can lead to a significant contribution towards reducing the effects of CO_2_ emissions from fossil fuels, which have been increasing during the last decades, especially in developing countries [5].

Nevertheless, microalgal biorefinery exploitation needs collective efforts from research and industry to improve economic viability and energy balance for the bioproduction of added-value products. The two main steps to tackle are cultivation and harvesting, so improvements have to be investigated to reduce high energy consumption, and total investment cost [5]. The choice of an appropriate target microorganism able to produce a multitude of added-value products is a critical starting point and can positively affect the probability of designing an economically sustainable process.

Astaxanthin, lutein, and β-carotene are valuable products which have an essential role in various fields of industrial interest. The high excitement surrounding these compounds is mainly due to their wide range of applications, properties, and market opportunities [[6], [7], [8]]. All these pigments can be produced through microalgal cutlivation [[9], [10], [11]]. *H.pluvialis* is a freshwater microalga studied since the second half of the 20th century, with particular emphasis on its carotenogenesis [12,13], which is still attracting both research and industrial interest. It can synthesise all of the three pigments mentioned above, and it is also able to grow autotrophically by using CO_2_ as carbon source. These two aspects make it attractive for both bioremediation and production of added-value compounds.

**Fig. 1:**
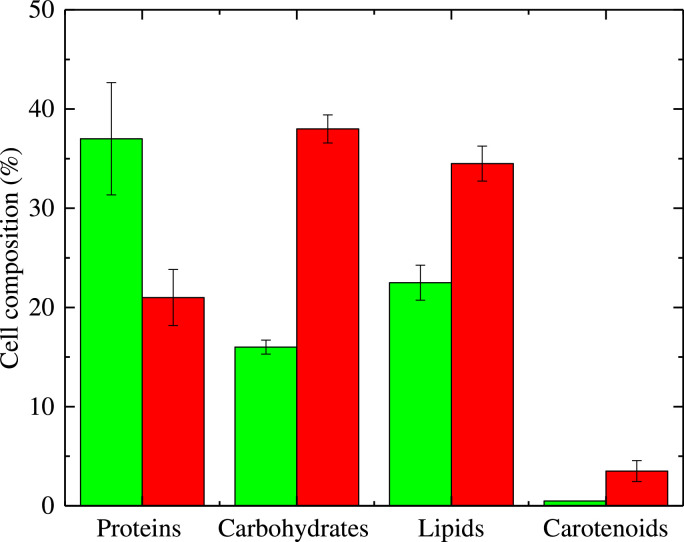
Cell composition differences between the green and red cultivation stage of *H. pluvialis* (data adapted from Shah (2016) [14]).

**Fig. 2:**
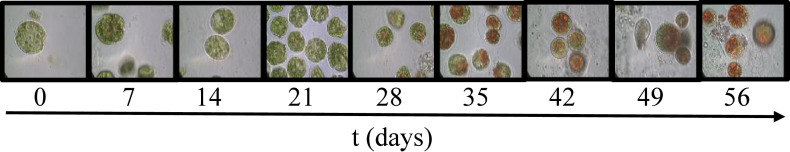
Light microscope images (magnificication 100X) of photoautotrophic cultivation during the base case (BC) experiment.

A particular feature of *H.pluvialis* cultivation is that it undergoes a transition under stress conditions, moving from a predominant protein cell content towards a prevalence of lipids, carbohydrates, and carotenoids, as schematically shown in Fig. 1. A phase where the cells are rapidly growing without stress can be referred to as the *green phase*, and the one where the cells are exposed to stress conditions can be referred to as the *red phase*. The differentiation above is mainly due to the cell colouration during the two phases, attributed to the green chlorophyll pigments during the growth and multiplication phase, and to astaxanthin during the stress phase. Consequently, the valuable pigments produced during the green phase are lutein and β-carotene, while the main pigment produced during the red phase is astaxanthin. Fig. 2 depicts the key morphological changes during the photoautotrophic cultivation of *H.pluvialis* as they transition from green to red phase.

The green phase of the cultivation takes place when the environmental conditions are “good enough” to favour cell growth and multiplication [15]. Stress conditions often lead to the formation of added-value products [16], and precise knowledge of these conditions can help in defining optimal production strategies. The *H.pluvialis* red phase has been linked to nutrient deprivation from lack of nitrogen, phosphorous, or sulphur. However, the mechanisms inducing the transition are not fully understood. The accumulation of carotenoids and lipids in this microalga seems to coincide with stress induction, and can lead to the simultaneous production of added-value products [[14], [17], [18], [19]].

Stress conditions seem to be a trigger for stopping cell multiplication; the cells start the process of lysis/death, and simultaneously the dry weight keeps increasing [20,21]. Hence, lysis/death can lead to loss of products in the extracellular media making their retrieval infeasible. Consequently, cell multiplication and cell number play an important role in the production of added-value products, as multiplication triggers cell phase transition. Cell number is vital as ideally as many cells as possible should undergo phase transition to enhance the production of added-value products. Mathematical models of various complexity and spanning different scales have been developed to describe the complex microalgal cultivation process [20]. Nevertheless, mathematical models that have been developed for *H.pluvialis* do not take into account lysis/death which can be responsible for product loss in the red phase where carotenoids accumulation takes place [22,23].

In this work, we construct a multi-scale model capable of predicting cell growth and lysis during the different cultivation phases of *H. pluvialis*. A segregated-structured model using a volume-structured population balance equation (PBE) coupled with a two-compartment structured model is proposed. The multi-scale model is able to predict cell density distribution, cell number, average cell volume, and extracellular and intracellular concentrations of nitrates (the form of nitrogen evaluated). Furthermore, it forms a solid basis for a modelling tool that can be extended to include more nutrients as well as product concentrations. The PBE internal coordinate is the volume which increases during the light period in the microalgae cell-cycle. Previous research has established that the volume is one of the variables involved in microalgae fission (cell division), including binary or multiple fission. Microalgal cells grow until they reach a commitment point after which they undergo fission even without light energy supply. Fission is considered to take place during the night [[24], [25], [26]]. In addition, our PBE-based model takes into account cell lysis relating it with cell dimension and nutrient depletion. The model parameters are fitted against various experiments with different nitrate concentrations and light levels, and are tested thoroughly through sensitivity analysis studies. The model prediction capabilities are subsequently examined against different environmental conditions.

## 2 Materials and methods

### 2.1 Strain and cultivation

The experimental campaign was performed through the cultivation of *Haematococcus pluvialis* strain FLOTOW (1844) CCAP 34/6 purchased from the Culture Collection of Algae and Protozoa (CCAP) Scotland, UK. The cells were maintained in photoautotrophic conditions by cultivating them in NIES-C (carbon-free) medium (rfs. Table 3) [27]. The microalgae were propagated prior to the experiments by inoculating them every 7 days in 200 mL of fresh NIES-C media at a constant concentration of 4240 cell mL^−1^. The 500 mL bottles containing the media were closed using porous sponge caps and placed on an orbital shaker at 130 rpm, and inside a growth cabinet at a constant top-side illumination of 60 µmol *m*^−2^ *s*^−1^ (16 h/8 h dark/light cycle) provided from day-light fluorescent tubes placed on the top of the cabinet. Hence, the light is considered to predominantly reach the cultivation perpendicularly to the top surface of the cultivation broth.

### 2.2 Lab-scale scale experiments

One of the main objectives of this work was to evaluate the effect of initial nitrates concentration, and incident light intensity on the growth of *H.pluvialis*, in terms of cell number and size, and to investigate the phenomena taking place over long cultivation times when nutrient depletion is prevalent. A total of five different experiments were carried out with different nitrate and light conditions. All of the experiments had a duration of 56 days, and a sacrificial sample was collected every 7 days. The NIES-C media [28] was used as a base case (BC) for the experiments (N BC). Nitrates were present in the form of $\text{Ca}{\left(NO_{3}\right)}_{2}4H_{2}O$, and $\text{KN}O_{3}$ giving a total nitrate content of 0.14 g *L*^−1^ for the BC. The media was modified to comprise (i) a low nitrogen case (N - -), decreasing by 50% the nitrate concentration of both nitogen sources, resulting in a total nitrate concentration of 0.07 g *L*^−1^ and (ii) a high nitrogen case (*N* ++) by increasing both nitrate sources by 50%, resulting in a total of 0.21 g *L*^−1^ of nitrates. All of the above experiments were carried out at 60 µmol *m*^−2^ *s*^−1^ light irradiance. The fourth experiment was carried out starting with the base case media concentrations, increasing the light intensity up to 200 µmol *m*^−2^ *s*^−1^ (*L* + +). Finally, the last experiment considered a 25% reduced nitrate concentration and a light intensity of 90 µmol *m*^−2^ *s*^−1^ (N -, *L* +). In order to have initial pH equal to 7 in all experiments, an appropriate quantity of 3M HCl was added after media preparation.

### 2.3 Analytical methods

#### 2.3.1 Cell number and size

Cell number was quantified using a Nexcelom Cellometer Auto T4 cell counter (Nexcelom Bioscience). The apparatus output gives the number histogram of the cells distributed in terms of size given the initial cell number. The average cell size was automatically calculated by the software provided.

#### 2.3.2 Nitrate analysis

Nitrate analysis was carried out by using a Metrohm ® 882 Compact IC plus. The apparatus allows measuring specific negative ions with the column Metrosep A Supp 5 150/4.0 mm. The standard was from Sigma-Aldrich, NO_3_^−^ ion solution at 1 g L^−1^, and it was diluted in order to be used in the range of interest.

## 3 The mathematical model

**Fig. 3:**
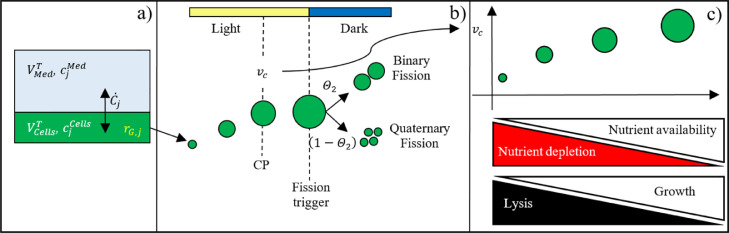
Main model features: (a). Intracellular and extracellular compartments, (b) growth and fission framework (adapted from Concas (2016)[29]), and (c) Cellular development behaviour based on nutrient availability.

A model able to predict microalgae growth and lysis, as well as the biosynthesis and accumulation of intracellular and extracellular compounds was constructed in this work. As shown in Fig. 3a, the reactor is divided in two volume compartments, an intracellular ($V_{Cells}^{T}$), and an extracellular ($V_{Med}^{T}$) one. Species concentrations in both compartments can vary due to factors such as the transport between the two compartments (${\dot{C}}_{j}$), and variation of the cell volume compartment. The intracellular compartment is where cell-level reactions take place, taken into account by a reaction term, $r_{G,j}$. In Section (3.2) a full description of the model equations for the extracellular and intracellular compartments is given. The intracellular volume, $V_{Cells}^{T}$, changes during the microalgae cultivation and the PBE in Eq. (1) allows us to quantify its variation, by taking into ccount the growth and fission process, schematically shown in Fig. 3b, and also the possibility of lysis/death taking place (Fig. 3c).

### 3.1 Population balance equation (PBE)

The PBE proposed in this work describes microalgae proliferation and lysis in a batch system by considering the volumetric growth of single cells G(v), the birth of new daughter cells B(v), the disappearance of mother cells M(v), and cell lysis, D(v) (Eq. (1)). The dynamic volume growth is considered to take place only during the light cycle phase of the cultivation. Hence, if the critical volume, $v_{c}$, is reached, cells can undergo fission even without light input during the dark phase of the cycle (Fig. 3b). At lower nutrient availability, the critical volume becomes lower and vice versa at high nutrient availability (Fig. 3c). The lysis phenomena are related to nutrient availability, being more likely to occur at low nutrients levels where the fission rate is sharply reduced due to nutrient depletion (Fig. 3c). Eq. (2), and Eq. (3) are the initial and the boundary conditions respectively for the PBE given by Eq. (1).

(1) $$\frac{\partial \Psi_{V}\left(v,t\right)}{\partial t}+G\left(v\right)=B\left(v\right)-M\left(v\right)-D\left(v\right)$$

(2) $$\Psi_{V}\left(v,t\right)={\Psi_{V}}^{0}\left(v\right)\;for\;t=0\;and\;\forall v$$

(3) $$\Psi_{V}\left(v,t\right)=0\;for\;t>0\;and\;v=0$$

Here, $\Psi_{V}$ *(v, t)* is the density distribution function (DBF) of the cell concentration at cell volume v and time t, homogeneously distributed in the reactor space. The continuous term G(v) in Eq. (4) represents the growth of single cells as a function of volume with rate $r_{V}$.

(4) $$G\left(v\right)=\frac{\partial \left(r_{V}\Psi_{V}\right)}{\partial v}$$

Based on the work presented in [30], the growth rate of a single cell represents the anabolic part of the metabolism proportional to the cell surface, which is represented as a function of volume through a simple mathematical manipulation (Eq. (5)). The volume growth rate is also a function of the two main limiting factors: the intracellular nitrate content (${C_{NO_{3}^{-}}}^{Int}$), and the average light intensity ($I_{Ave}$) in the reactor.

(5) $$r_{V}\left(\;I_{ave},{C_{NO_{3}^{-}}}^{Int},v\right)=\mu_{max}\cdot f_{{C_{NO_{3}^{-}}}^{Int}}\left({C_{NO_{3}^{-}}}^{Int}\right)\cdot f_{I_{Ave},G}\left(I_{Ave}\right)\cdot {\left(\frac{3}{4\pi }\right)}^{\frac{2}{3}}v^{2/3}$$

Here $\mu_{max}$ is the maximum specific growth rate. The kinetics for internal nitrogen evolution is given by a double affinity kinetic law (Eq. (6)), which takes into account how cells activate different mechanisms when exposed to high or low intracellular nitrates concentration.

(6) $$f_{{C_{NO_{3}^{-}}}^{Int}}=\left(1-x_{A}\right)\cdot f_{{C_{NO_{3}^{-}}}^{Int},L}+x_{A}\cdot f_{{C_{NO_{3}^{-}}}^{Int},H}$$

The term $x_{A}$ represents the affinity of the cell growth for high levels of intracellular nitrates, whereas $\left(1-x_{A}\right)$ indicates the affinity for low levels of intracellular nitrates. Both low-, $f_{{C_{NO_{3}^{-}}}^{Int},L}$ (Eq. (7)) and high-affinity $f_{{C_{NO_{3}^{-}}}^{Int},H}$ (Eq. (8)) nitrates kinetic functions contain an inhibition term which is a modification of the one proposed by [31], allowing for inhibition to take place when nitrates reach high concentrations [32,33].

(7) $$f_{C_{NO_{\bar{3}}}^{Int},L}=\frac{{\left(C_{NO\bar{{}_{3}}}^{Int}\right)}^{n_{L}}}{{\left(K_{C_{NO\bar{{}_{3}}}^{Int},LG}\right)}^{n_{L}}+{\left(C_{NO\bar{{}_{3}}}^{Int}\right)}^{n_{L}}+{\left(\frac{{\left(C_{NO\bar{{}_{3}}}^{Int}\right)}^{2}}{K_{C_{NO\bar{{}_{3}}}^{Int},L:I}}\right)}^{n_{L}}}$$

(8) $$f_{C_{NO_{\bar{3}}}^{Int},H}=\frac{{\left(C_{NO\bar{{}_{3}}}^{Int}\right)}^{n_{H}}}{{\left(K_{C_{NO\bar{{}_{3}}}^{Int},H}\right)}^{n_{H}}+{\left(C_{NO\bar{{}_{3}}}^{Int}\right)}^{n_{H}}+{\left(\frac{{\left(C_{NO\bar{{}_{3}}}^{Int}\right)}^{2}}{K_{C_{NO\bar{{}_{3}}}^{Int},H:I}}\right)}^{n_{H}}}$$

Here, ${C_{NO_{3}^{-}}}^{Int}$ is the concentration of internal nitrates, $K_{{C_{NO_{3}^{-}}}^{Int},LG}$ and $K_{{C_{NO_{3}^{-}}}^{Int},L:I}$ are saturation and inhibition coefficients for the low affinity case, and $K_{{C_{NO_{3}^{-}}}^{Int},H}$ and $K_{{C_{NO_{3}^{-}}}^{Int},H:I}$ are saturation and inhibition constants for the high-affinity case, respectively, and $n_{L}$, $n_{H}$ are shape factors. Eq. (6), therefore, represents a linear combination of the low- and high-affinity kinetics with $x_{A}$ being a fitting parameter, $x_{A}\in \left[0,1\right],\;$representing the affinity level for low and high nitrates concentration.

The light kinetics (Eq. (9)) involved in the single-cell volume growth considers an Andrew modified law, taking into account the average light intensity, $I_{Ave}$,

(9) $$f_{I_{Ave},G}=\frac{{I_{Ave}}^{n_{I}}}{{K_{I_{Ave}}}^{n_{I}}+{I_{Ave}}^{n_{I}}+{\left(\frac{{I_{Ave}}^{2}}{K_{I_{Ave}:I}}\right)}^{n_{I}}}$$

$K_{I_{Ave}}$ being the light saturation constant, $K_{I_{Ave}:I}$ the light inhibition constant, and $n_{I}$ a shape factor. The average light intensity is calculated through the Lambert-Beer law considering light perpendicularly hitting the reactor surface:

(10) $$I_{ave}=\frac{I_{0}}{\beta \cdot \rho_{Cells}\cdot \frac{V_{Cells}^{T}}{V_{R}^{T}}\cdot Z}\cdot \left[1-e^{-\beta \cdot \rho_{Cells}\cdot \frac{V_{Cells}^{T}}{V_{R}^{T}}\cdot Z}\right]$$

where $I_{0}$ is the incident light, Z the depth of the vessel, β the absorption coefficient, and the global term, $\rho_{Cells}\frac{V_{Cells}^{T}}{V_{R}^{T}}$, is the biomass concentration expressed as a function of the cell density ($\rho_{Cells}$), the total cell volume ($V_{Cells}^{T}$), and the reactor volume ($V_{R}^{T}$). The cell density is assumed to be constant through the cultivation time [34].

M(v) in Eq. (11) is a sink term representing cell birth, and it expresses how mother cells with volume $v^{\prime }$disappear due to a birth event.

(11) $$M\left(v\right)=\Gamma^{f}\left(v,{C_{NO_{3}^{-}}}^{Int},I_{av}\right)\cdot \Psi_{V}\left(v^{\prime }\right)$$

Here $\Gamma^{f}$ is the transition rate given by Eq. (12):

(12) $$\Gamma^{f}\left(v,{C_{NO_{3}^{-}}}^{Int},I_{av}\right)=r_{f}\left(\;{C_{NO_{3}^{-}}}^{Int},v\right)\cdot \gamma^{f}\left(v\right)\cdot SF\left(I_{av}\right)$$

$r_{f}\left(\;{C_{NO_{3}^{-}}}^{Int},v\right)$ being the kinetic dependence of the transition rate on the limiting nutrient (internal nitrates), which takes the form presented in Eq. (14); $\gamma^{f}\left(v\right)$ is a gamma function of the nutrients contained in the extracellular and intracellular compartments presented in Eq. (17)) and $SF(I_{av})$ a switch factor reported in Eq. (13), which ensures that $\Gamma^{f}$ has a real positive value exclusively during the night time.

(13) $$SF\left(I_{ave}\right)=\{\begin{array}{c} SF=1\;\;I_{ave}=0\\ SF=0\;I_{ave}>0 \end{array}$$

(14) $$r_{f}\left(C_{NO\bar{{}_{3}}}^{Int},v\right)=\mu_{max}\cdot f_{c_{NO\bar{{}_{3}},0}^{Med}}\left(c_{NO\bar{{}_{3}},0}^{Med}\right)\cdot f_{I_{0}}\left(I_{0}\right)\cdot f_{C_{NO\bar{{}_{3}}}^{Int}}\left(C_{NO\bar{{}_{3}}}^{Int}\right)\cdot {\left(\frac{3}{4\pi }\right)}^{\frac{2}{3}}v^{2/3}$$

In Eq. (14) the internal nitrates function $f_{{C_{NO_{3}^{-}}}^{Int}}\left({C_{NO_{3}^{-}}}^{Int}\right)$ has the same form as the one for the volume growth rate presented in Eq. (6). $f_{c_{NO_{3}^{-,0}}^{Med}}\left(c_{NO_{3}^{-,0}}^{Med}\right)$ and $f_{I_{0}}\left(I_{0}\right)$, given in Eq. (15) and Eq. (16), respectively, are S-shape functions of the initial extracellular nitrates and the incident light, and they express the inhibition effect of both parameters on microalgae fission.

(15) $$f_{c_{NO_{3}^{-,0}}^{Med}}\left(c_{NO_{3}^{-,0}}^{Med}\right)=\frac{1}{1+exp^{\left[\left(c_{NO_{3}^{-,0}}^{Med}-hf_{1}\right)/hs_{1}\right]}}$$

(16) $$f_{I_{0}}\left(I_{0}\right)=\frac{1-h_{min}}{1+exp^{\left[\left(I_{0}-hf_{2}\right)\;/\;hs_{2}\right]}}$$

Here $hf_{1}$ and $hf_{2}$ are flex points of the corresponding functions, and $hs_{1}$, $hs_{2}$ are shape factors. The incident light function (Eq. (16)) has minimum value ($h_{min}$) representing the maximum inhibition achievable for high light concentration.

A particular feature of this model is to describe $\gamma^{f}\left(v\right)$ (Eq. (17)) as a function of the nutrients contained in the extracellular and intracellular compartments.

(17) $$\gamma^{f}\left(v\right)=\frac{f_{V}\left(v\right)}{1-\int_{0}^{v}f_{V}\left(v^{\prime }\right)dv{}^{\prime }}$$

Here $f_{V}\left(v\right)$ is a gaussian shape function (Eq. (18)) with variance $\sigma_{c}$, and an average critical volume $v_{c}$.

(18) $$f_{V}\left(v\right)=\frac{1}{\sqrt{2\pi \sigma_{c}^{2}}}exp^{\left[-\frac{1}{2}{\left(\frac{v-v_{c}}{\sigma_{c}}\right)}^{2}\right]}$$

As can be seen in Eq. (19), the critical volume is not constant, but is a function of the extracellular and intracellular nitrates concentration. The (fitted) coefficient, $y_{A}\in \left[0,1\right]$ indicates how the kinetic contributions are split between external and internal nitrate functions.

(19) $$v_{c}=v_{c,max}\left[y_{A}\cdot f_{{C_{NO_{3}^{-}}}^{Ext},V_{c}}\left({C_{NO_{3}^{-}}}^{Ext}\right)+\left(1-y_{A}\right)\cdot f_{{C_{NO_{3}^{-}}}^{Int},V_{c}}\left({C_{NO_{3}^{-}}}^{Int}\right)\right]$$

Two Monod [35] functions were adopted for extracellular (Eq. (20)) and intracellular (Eq. (21)) concentration, considering that the critical volume changes follow these laws:

(20) $$f_{{C_{NO_{3}^{-}}}^{Ext},V_{c}}\left({C_{NO_{3}^{-}}}^{Ext}\right)=\frac{{C_{NO_{3}^{-}}}^{Ext}}{K_{{C_{NO_{3}^{-}}}^{Ext},V_{c}}+{C_{NO_{3}^{-}}}^{Ext}}$$

(21) $$f_{{C_{NO_{3}^{-}}}^{Int},V_{c}}\left({C_{NO_{3}^{-}}}^{Int}\right)=\frac{{C_{NO_{3}^{-}}}^{Int}}{K_{{C_{NO_{3}^{-}}}^{Int},V_{c}}+{C_{NO_{3}^{-}}}^{Int}}$$

$K_{{C_{NO_{3}^{-}}}^{Ext},V_{c}}$ and $K_{{C_{NO_{3}^{-}}}^{Int},V_{c}}$ are saturation constants for the extracellular and intracellular nitrate functions, respectively.

The term B(v) in Eq. (1), reported in Eq. (22), represents the birth of microalgae by multiple fission, where a mother cell with volume $v^{\text{'}\;\;}$can generate two or four daughter cells, hence the *i* index, which can be equal to 2 or 4. This expression is adapted from the first work, where the multiple fission term for microalgae PBE models was presented [29]. $\Theta_{i}$ is the probability for the fission event *i*, hence $\Theta_{2}$ and $\Theta_{4}$ are the probabilities for fission events generating two and four daughters, respectively.

(22) $$B\left(v\right)=\sum_{i=2,4}i\Theta_{i}\int_{v}^{\infty }\Gamma^{f}\left(v,C_{TN}^{Med}\right)P_{i}\left(v,v^{\prime }\right)\Psi_{V}\left(v^{\prime },t\right)dv^{\prime }$$

The partitioning continuous distribution function respects the condition of normalization (Eq. (23)) postulated by Fredrickson et al. [36].

(23) $$\int_{o}^{v^{\prime }}p_{i}\left(v,v^{\prime }\right)dv=1$$

$p_{i}\left(v,v^{\prime }\right)\;$given in Eq. (24) belongs to the beta family functions, and it is a generalized Hill-Ng distribution function [37]; $\alpha_{i}$ and $\delta_{i}$ are specific parameters for the distribution which must satisfy the condition in Eq. (25) [38]; $\beta (\alpha_{i},\delta_{i})$ is a class beta function adapted for multiple fission as shown in [29].

(24) $$p_{i}\left(v,v^{\prime }\right)=\frac{1}{\beta \left(\alpha_{i},\delta_{i}\right)}\frac{1}{v^{\prime }}{\left(\frac{v}{v^{\prime }}\right)}^{\alpha_{i}}{\left(1-\frac{v}{v^{\prime }}\right)}^{\delta_{i}}\;i=2,\;4$$

(25) $$\delta_{i}=\alpha_{i}\left(i-1\right)$$

The main issue with this kind of distribution is to maintain the normalization condition of Eq. (23), so the normalised version of Eq. (24) is proposed:

(26) $$P_{i}\left(v,v^{\prime }\right)=\frac{p_{i}\left(v,v^{\prime }\right)}{\int_{o}^{v{}^{\prime }}p_{i}\left(v,v^{\prime }\right)dv}$$

The lysis term, D(v), in Eq. (1) includes a lysis rate, $L(v,{C_{NO_{3}^{-}}}^{Int})$ and the density function, and is considered to be a first-order process (Eq. (27)).

(27) $$D\left(v\right)=L\left(v,{C_{NO_{3}^{-}}}^{Int}\right)\cdot \Psi_{V}\left(v,t\right)$$

The lysis rate is given in Eq. (28) and has an exponential form, including a pre-exponential factor $k_{Lys,0}$, and a negative exponential term where a parameter $k_{Lys,S}$ is multiplied by the fission rate, $r_{f}\left(\;{C_{NO_{3}^{-}}}^{Int},v\right)$.

(28) $$L\left(v,{C_{NO_{3}^{-}}}^{Int}\right)=k_{Lys,0}e^{\left[-k_{Lys,S}\cdot r_{f}\left(\;{C_{NO_{3}^{-}}}^{Int},v\right)\right]}$$

Eq. (28) expresses how lysis increases when the fission phenomena slow down, and the cells are more likely to undergo disruption.

### 3.2 The extracellular and intracellular compartments

Fig. 3a shows the subdivision of the reactor volume. The total reactor volume $V_{R}^{T}$ is the sum of the total intracellular cell volume $V_{Cells}^{T}$ and the total extracellular media volume $V_{Med}^{T}$.

(29) $$V_{R}^{T}=V_{Cells}^{T}+V_{Med}^{T}$$

The total volume of the cells is the 1st order moment of the cell population density $\Psi_{V}\left(v,t\right)$. A significant advantage of utilising two compartments is to allow the use of two intrinsic concentrations in the media and in the cell compartments, defined as $m_{j}^{\text{Med}}/V_{\text{Med}}^{T}$ and $m_{j}^{\text{Cells}}/V_{\text{Cell}}^{T}$, respectively [39]. The material balances for the extracellular (Eq. (30)), and the intracellular compartment (Eq. (31)) consider the extracellular and intracellular volume, respectively. The equation for the extracellular compartment considers a dilution term that includes the change of cell volume in time, $\frac{dV_{Cells}^{T}}{dt},$ and a mass transfer term ${\dot{C}}_{j}$, which describes the mass transport of the j^th^ species between the two compartments.

(30) $$\frac{dc_{j}^{Med}}{dt}=\frac{1}{V_{R}^{T}-V_{Cells}^{T}}\left(c_{j}^{Med}\frac{dV_{Cells}^{T}}{dt}-{\dot{C}}_{j}\right)\;j=NO_{3}^{-}$$

Eq. (31) is developed in an analogous way to Eq. (30), with the mass transfer term having the opposite sign, signifying that mass disappearing from the extracellular compartment is appearing in the intracellular compartment and vice versa. Moreover, the time derivative of the total cell volume also has the opposite sign in Eq. (31). A positive derivative will lead to an increase in the intrinsic extracellular concentration and vice versa in the case of intracellular mass balance.

(31) $$\frac{dc_{j}^{Cells}}{dt}=r_{G,j}+\frac{1}{V_{Cells}^{T}}\left({\dot{C}}_{j}-c_{j}^{Cells}\frac{dV_{Cells}^{T}}{dt}\right)\;j=NO_{3}^{-}$$

The material transport between the two compartments term is given by Eq. (32):

(32) $${\dot{C}}_{j}={\dot{C}}_{NO_{3}^{-}}=\rho_{U,Max}\cdot \frac{C_{NO_{3}^{-}}^{Ext}}{K_{C_{NO_{3}^{-}}^{Ext},U}+C_{NO_{3}^{-}}^{Ext}}\cdot 4\pi {\left(\frac{3}{4\pi }\right)}^{\frac{2}{3}}V_{Cell}^{T\;2/3}$$

Here $\rho_{U,Max}$ indicates the maximum specific uptake rate for nitrates, and $K_{{C_{NO_{3}^{-}}}^{Ext},U}$ the saturation constant of the uptake Monod function. The last right hand-side term represents the total surface of the cell membrane, which depends on the amount of channels/transport proteins on the cell membrane. The nitrate reaction consumption rate, $r_{G,NO_{3}^{-}}$, is present only in the intracellular material balance Eq. (31), and depends on the consumption rate presented in Eq. (33).

(33) $$\begin{array}{c} r_{G,NO_{3}^{-}}=\rho_{C,Max}\cdot \frac{{\left(C_{NO_{3}^{-}}^{Int}\right)}^{n_{c}}}{{\left(K_{C_{NO_{3}^{-}}^{Int},c}\right)}^{n_{c}}+{\left(C_{NO_{3}^{-}}^{Int}\right)}^{n_{c}}+{\left(\frac{{\left(C_{NO_{3}^{-}}^{Int}\right)}^{2}}{K_{C_{NO_{3}^{-}}^{Int},C:I}}\right)}^{n_{c}}}\\ \;\;\;\cdot \frac{I_{Ave}^{n_{Ic}}}{K_{I_{Ave,c}^{n_{Ic}}}+I_{Ave}^{n_{Ic}}+{\left(\frac{I_{Ave}^{2}}{K_{I_{Ave},c:I}}\right)}^{n_{Ic}}} \end{array}$$

The expression includes two Andrew modified laws for the kinetics of consumption, which take into account that (a) high nitrates concentration can inhibit nitrate consumption and (b) the average light has a similar effect, where nitrates consumption is favoured until the light reaches a certain level after which it is negatively influenced. $\rho_{C,Max}$ is the maximum nitrate consumption rate, $K_{{C_{NO_{3}^{-}}}^{Int},c}$ and $K_{I_{Ave,c}}$ are saturation coefficients, and $K_{{C_{NO_{3}^{-}}}^{Int},C:I}$ and $K_{I_{Ave},c:I}$ are inhibition coefficients for nitrate and light respectively. Finally, $n_{c}$ and $n_{Ic}$ are shape factors for the nitrate and light functions.

### 3.3 Parameter fitting and sensitivity analysis

The model presented in Eqs. (1) to (33) consists of one partial-integro differential equation (PIDE) to describe the intracellular compartment, and two ordinary differential equations (ODEs) to represent the extracellular compartment. The model includes a parameter set P consisting of 34 parameters, which are estimated through fitting to a range of experiments. All estimated parameters are given in Table 1, while additional parameters obtained from the literature or from other calculations are shown in Table 2. Parameters are estimated by minimising the error between the vector of state variables of the model outputs, $y_{i,j}^{Mod}\left(P\right)\in R^{NV},$ and the vector of variables of the experimental measurements $y_{i,j}^{Exp}\left(P\right)\in R^{NV}$, *NV* being the number of experimentally measured variables, for a number of experiments *j* = 1, NExp and time sampling points *i* = 1, Ntj. The corresponding objective function, Obj(P), is given by:

(34) $$Obj\left(P\right)=\sum_{j=1}^{NExp}\sum_{i=1}^{Ntj}{\left[y_{i,j}^{Exp}-y_{i,j}^{Mod}\left(P\right)\right]}^{T}W_{i,j}^{-1}\left[y_{i,j}^{Exp}-y_{i,j}^{Mod}\left(P\right)\right]$$

**Table 1:** Model parameter values.

Parameter n°	Symbol	Parameter description	Value M34	Value M29	Unit
1	β	Light attenuation coefficient	1.44 × 10^1^	1.64 × 10^1^	L *g*^−1^*m*^−1^
2	$\mu_{\max}$	Maximum specific growth rate	0.45 × 10°	0.41 × 10°	µm *h*^−1^
3	$k_{Lys,0}$	Pre-exponential factor cell lysis	1.75 × 10^−3^	1.46 × 10^−3^	*h*^−1^
4	$k_{Lys,S}$	Exponential factor cell lysis	0.51 × 10°	–	h µm^−3^
5	$\rho_{U,Max}$	Maximum rate nitrates uptake	7.72 × 10^−2^	7.53 × 10^−2^	g µm^−2^h^−1^
6	$K_{{C_{NO_{3}^{-}}}^{Ext},U}$	Saturation constant nitrates uptake	5.44 × 10^−2^	5.19 × 10^−2^	g *L*^−1^
7	$\Theta_{2}$	Probability of two daughter cells birth per mitotic event	5.29 × 10^−1^	4.72 × 10^−2^	–
8	$K_{{C_{NO_{3}^{-}}}^{Int},LG}$	Saturation constant nitrate low-affinity growth and fission	2.85 × 10^−4^	2.87 × 10^−4^	g *L*^−1^
9	$K_{{C_{NO_{3}^{-}}}^{Int},L:I}$	Inhibition constant nitrate low-affinity growth and fission	6.90 × 10^−5^	8.74 × 10^−5^	g *L*^−1^
10	$n_{L}$	Shape factor nitrate low-affinity growth and fission	0.87 × 10°	1.08 × 10°	–
11	$K_{{C_{NO_{3}^{-}}}^{Int},H}$	Saturation constant nitrate high-affinity growth and fission	1.97 × 10^1^	1.54 × 10^1^	g *L*^−1^
12	$K_{{C_{NO_{3}^{-}}}^{Int},H:I}$	Inhibition constant nitrate high-affinity growth and fission	1.08 × 10^3^	–	g *L*^−1^
13	$n_{H}$	Shape factor nitrate high-affinity growth and fission	6.88 × 10^−1^	6.06 × 10^−1^	–
14	$x_{A}$	Affinity factor nitrate growth and fission	6.65 × 10^−1^	5.98 × 10^−1^	–
15	$K_{I_{Ave}}$	Saturation constant average light growth and fission	3.36 × 10^1^	4.24 × 10^1^	µmol *m*^−2^s^−1^
16	$K_{I_{Ave}:I}$	Inhibition constant average light growth and fission	2.32 × 10^2^	2.90 × 10^2^	µmol *m*^−2^s^−1^
17	$n_{I}$	Shape factor average light growth and fission	7.86 × 10^−1^	9.00 × 10^−1^	–
18	$K_{{C_{NO_{3}^{-}}}^{Int},C}$	Saturation constant nitrate consumption	6.80 × 10^1^	5.51 × 10^1^	g *L*^−1^
19	$K_{{C_{NO_{3}^{-}}}^{Int},C:I}$	Inhibition constant nitrate consumption	2.88 × 10^2^	2.82 × 10^2^	g *L*^−1^
20	$n_{c}$	Shape factor nitrate consumption	1.13 × 10°	1.13 × 10°	–
21	$K_{I_{Ave,c}}$	Saturation constant average light consumption	5.44 × 10^1^	6.08 × 10^1^	µmol *m*^−2^s^−1^
22	$K_{I_{Ave},c:I}$	Inhibition constant average light consumption	7.39 × 10^2^	–	µmol *m*^−2^s^−1^
23	$n_{Ic}$	Shape factor average light consumption	1.20 × 10°	1.01 × 10°	–
24	$\rho_{C,Max}$	Maximum rate of nitrate consumption	2.28 × 10^1^	1.96 × 10^1^	g *h*^−1^*L*^−1^
25	$v_{c,max}$	Maximum critical volume	1.21 × 10^4^	1.10 × 10^4^	µm^3^
26	$y_{A}$	Affinity factor critical volume	2.29 × 10^−1^	–	–
27	$K_{{C_{NO_{3}^{-}}}^{Ext},V_{c}}$	Saturation constant external nitrate critical volume	5.91 × 10^−2^	–	g *L*^−1^
28	$K_{{C_{NO_{3}^{-}}}^{Int},V_{c}}$	Saturation constant internal nitrates critical volume	5.31 × 10°	6.11 × 10°	g *L*^−1^
29	$hf_{1}$	Flex point initial nitrates fission inhibition	1.90 × 10^−1^	1.72 × 10^−1^	g *L*^−1^
30	$hs_{1}$	Shape factor initial nitrates fission inhibition	3.90 × 10^−2^	4.80 × 10^−2^	g *L*^−1^
31	$hf_{2}$	Flex point incident light fission inhibition	5.03 × 10^1^	4.32 × 10^1^	µmol *m*^−2^s^−1^
32	$hs_{2}$	Shape factor incident light fission inhibition	1.32 × 10^1^	1.53 × 10^1^	µmol *m*^−2^s^−1^
33	$h_{min}$	Minimum fission factor for incident light	4.37 × 10^−1^	4.73 × 10^−1^	–
34	$\sigma_{c}$	Standard deviation critical distribution	3.37 × 10^3^	3.37 × 10^3^	µm^3^

**Table 2:** Literature and measurable parameters.

	Parameter description	Value	Unit	Refs.
$\alpha_{2}$	Hill-Ng distribution 1st parameter, 2 daughters	4.00 × 10^1^	–	[29]
$\;\alpha_{4}$	Hill-Ng distribution 1st parameter, 4 daughters	1.34 × 10^1^	–	[29]
$\;\delta_{2}$	Hill-Ng distribution 2nd parameter, 2 daughters	4.00 × 10^1^	–	[29]
$\;\delta_{4}$	Hill-Ng distribution 2nd parameter, 4 daughters	4.00 × 10^1^	–	[29]
$\;\sigma_{0}$	Standard deviation initial distribution	2.31 × 10^3^	–	This work (Initial Distribution Fitting)
$\mu_{0}$	Mean initial value distribution	6.94 × 10^3^	µm^3^	This work (Initial Distribution Fitting)
$\rho_{\mathrm{Cells}}$	Cell average density	1.09 × 10^−12^	g µm^−3^	(T. [34])
$V_{R}^{T}$	Culture volume	2.15 × 10^2^	mL	This work (Measurable parameter)
Z	Culture depth	5.00 × 10^−2^	m	This work (Measurable parameter)

**Table 3:** NIES-C media composition.

Species	Concentration (g L^−^^1^)
$Ca{\left(NO_{3}\right)}_{2}4H_{2}O$	0.225
$KNO_{3}$	0.15
$\beta -Na_{2}glycerophosphate\;5H_{2}O$	0.05
$MgSO_{4}7H_{2}O$	0.04
Vitamin $B_{12}$	0.000001
Biotin	0.000001
Thiamine HCl	0.0001
Tris (hydroxymethyl)aminomethane	0.5
$FeCl_{3}6H_{2}O$	0.003
$Na_{2\;}EDTA\;2H_{2}O$	0.000588
$MnCl_{2}4H_{2}O$	0.000108
$ZnSO_{4}7H_{2}O$	0.000066
$CoCl_{2}6H_{2}O$	0.000012
$Na_{2}MoO_{4}2H_{2}O$	0.0000075

We use a combination of stochastic and deterministic optimisation to minimise Obj(P),namely a genetic algorithm, exploiting the *ga* function in Matlab with multiple restarts to obtain a family of solutions around the (global) optimum and nonlinear programming (NLP) using *fmincon* function in Matlab to pinoint the actual optimum solution. The sparse magnitude of the fitting variables suggests the use of weights ($W_{i,j}$) in the objective function. Specifically, the weight matrix $W_{i,j}\;$ is diagonal consisting of the values of the *NV* experimentally measured variables for the *i*^th^ sampling time and the *j*^th^ experiment.

(35) $$W_{i,j}=diag\left({\left(y_{1}^{Exp}\left(t_{i,j}\right)\right)}^{2},\ldots ,{\left(y_{NV}^{Exp}\left(t_{i,j}\right)\right)}^{2}\right)$$

Extensive sensitivity analysis was subsequently performed to reduce parameter space by identifying the least sensitive parameters. The following expression is the normalized local sensitivity for the *m*^th^ parameter and the *n*^th^ variable at time t. Sensitivities are normalized with respect to the initial value of the parameter $P_{m}$, and the initial value of the variable $s_{n}$ around which the linearization is carried out.

(36) $$S_{nm}\left(\hat{P},t\right)=\frac{\partial s_{n}}{\partial P_{m}}\frac{P_{m}}{s_{n}}\left(t\right)$$

A concatenated matrix containing all the normalised sensitivities is constructed as shown in previous research [40]. The matrix has the same number of columns as the number of parameters. The number of rows, nrows, is calculated as shown in Eq. (37), where $Nvar_{j}$ is the number of variables for the j^th^ experiment.

(37) $$nrows=\sum_{j}^{NExp}Nvar_{j}Nt_{j}$$

The L2 norm for each column was calculated by using the *norm* function in Matlab, obtaining a vector with size equal to the number of parameters, its elements ranked in ascending order. Through sensitivity analysis the 34 parameters of the model were reduced to 29 as discussed in the next section.

## 4 Results and discussion

### 4.1 Experimental results

**Fig. 4:**
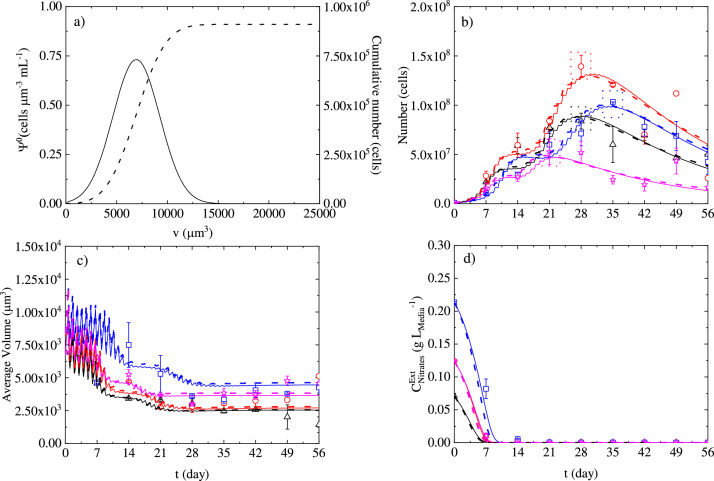
(a) Initial density distribution (solid line), and initial cell cumulative number (dashed line) for all experiments. (b) Cell number vs time (days). Experimental data (symbols), 34P model fitting (solid lines), 29P model fitting (dashed lines) (c) average cell volume (d) extracellular nitrates. Black triangles and solid/dashed lines N(–) L(BC), red circles and solid/dashed lines N(BC) L(BC), blue squares and solid/dashed lines N(++) L(BC), magenta stars and solid/dashed lines N(BC) L(++)).

The results obtained from different experiments carried out as explained in Section 2.2 show particular features for all the different runs. Generally, a cultivation time of around 28 days is where the highest cell number (calculated as the 0th order moment of the cell density multiplied by the total reactor volume, Appendix B, Eq. B1) is achieved, excluding the case with high initial nitrates concentration (*N*++) in which the peak seems to be approximately around 35 days. As shown in Fig. 4b, the initial nitrates content of 0.14 g *L*^−1^, N(BC), leads to the highest maximum cell number, after which, increasing nitrates concentration to 0.21 g *L*^−1^ (*N*++), leads to a decrease of the maximum cell number by about 26%. As also depicted in Fig. 4b, incident light of 200 µmol *m*^−2^s^−1^ (*L*++) strongly inhibits cell growth, leading to a decrease of the maximum cell number value of around 45%, compared with the base case scenario. The obtained cell number maximum values are of the same order of magnitude with comparable works in the literature for photoautotrophic cultivation, despite the fact that all the systems in the literature have air or air/CO_2_ mixture feeding. Only two cases in the literature are comparable with our experiments in terms of nitrogen content, and they both exhibited a max cell number of about 46% and 61% lower than our experiments, respectively [41,20]. The rest of the systems in the literature have a higher nitrogen content of 1.09 g *L*^−1^, and the highest cell content without considering enhancing multistage strategies is lower by 7% to 74% respectively [42,18] compared to our base case with a maximum cell number of 6.46 × 10^5^ cells mL^−1^.

A single microalgae cell grows in size until the achievement of a commitment point after which it undergoes fission if exposed to darkness [24,43,25]. Hence, the expectation in terms of cell average volume (calculated as the 1st order moment of the cell density divided by the 0th order moment of the cell density, Appendix B, Eq. B2) should be a rapid adaptation, after some multiplication cycles, to a steady average size also called balanced growth. The initial average size was the same for all the experiments, as the inoculum always came from a 7-day base case (BC) experiment in terms of nitrates concentration (N) and incident light intensity (L), (N (BC) L (BC)). Fig. 4c illustrates that there is a steady and gradual decrease in the average cell volume during the growth stage. We can also see that the average cell volume is generally lower in the case with the lowest initial nitrates concentration (N (–) L (BC)), and increases as the initial nitrate concentration increases. As depicted in Fig. 4c, at late cultivation stages the average cell volume will decrease, by approximately 75% in the case of N (–) L (BC), compared to the initial value (8412 µm^3^), while in the base case N (BC) L (BC) the cell volume decreases by approximately 56%, and in the case of N (++) L (BC) by around 52%. This indicates a significant variation of the average cell volume in the final stages of growth compared with the values at time zero. The high incident light experiment N (BC) L (++) reveals a higher average cell volume compared with the base case scenario, and the trend is both during the growth phase (up to day 30) and beyond. Predominantly during the first stages of the cultivation, for all of the experiments, oscillations of the average cell volume can be observed (Fig. 4c). They represent the oscillatory growth of the cells in terms of average size due to the light/dark cycles they are exposed to. During the light cycles, cells grow in terms of size, but they do not undergo fission. On the contrary, cells undergo fission during the dark cycle(s), which leads to an increase in the number of daughter cells, causing a decrease to the average volume of the cell population.

In is worthwhile to mention that the strict correlation between cell size and nutrient levels discussed in the literature involves mainly yeast cells, showing that nutrient-rich media induces bigger cell sizes, and nutrient-poor media leads to smaller cell sizes [44]. The hypothesis is that cells in an abundant nitrogen environment grow more in the G2/M phase of the cell cycle, consequently leading to larger daughter cells, and to a larger average cell size during growth [45,46]. The lysis phase as depicted in Fig. 4b takes place during the transition of cells into the red stage (cf. Fig 1). The cell number values at day 56, are smaller than the maximum values achieved during the growth phase, due to lysis. The cell loss fraction is between 0.47, and 0.83 for the different experiments, compared to the cell number at day 56, with the maximum cell number value achieved during the growth phase. In this view, cell biomass does not follow the same trend as the cell number. When cell lysis starts taking place, the content of biomass measured by conventional methods such as dry cell weight does not decrease like the cell number does, but it keeps increasing or it reaches a steady value (data not shown). The measurements of dry cell weight were considered in this case as biased by debris and residuals of the cell lysis, which were considerable taking into account the cell loss fractions obtained by direct image measurements. Due to these considerations, the cell number measurements were considered more reliable than biomass measurements.

As shown in Fig. 4d, extracellular nitrates are fully depleted in all of the experiments between the 7th and the 21st day of cultivation. Specifically, the nitrates concetration drops to zero before seven days for N (–) L (BC), between 7 and 14 days for N (BC) L (BC), N (BC) L (++), and N (-) L (+), and finally between 14 and 21 days for the case with the highest initial nitrates content N (++) L (BC). However, the cell number growth continues beyond day 21 in all experiments, indicating the ability for microalgae to store nitrates, or more generally nitrogen, as it is needed for maintaining the cell metabolism. The above phenomena have been also widely demonstrated for phytoplankton and diatoms [[47], [48], [49]].

### 4.2 Model fitting

One important purpose of the model was to be able to accurately describe the nutrient depletion phase including cell lysis during the late stage of cultivation, as that phase is the one where cells are more likely to give added-value products. The initial density distributions of the microalgae cells (${\Psi_{V}}^{0}\left(v\right)$) were considered as a Gaussian shape function, and were derived from an experimental histogram fitting (data not shown). The initial density distribution is shown in Fig 4a and is the same for all the fitting and prediction simulations, based on the fact that the inoculum at time zero always comes from an experiment at day 7 with N (BC) L (BC) conditions. The initial cell number is 911,400, as the cumulative number reveals in Fig. 4a. Fig. 4b–d, which show the fitting results for four different experiments with three different initial nitrogen concentrations (N (–) L (BC), N (BC) L (BC), N(++) L (BC)), and two different incident light intensities (N (BC) L (++)). Moreover, these figures depict the fitting for both the cases of the model with 34 and 29 parameters, respectively. The parameter number reduction from 34 to 29 was possible by carrying out a sensitivity analysis, which is a crucial tool to evaluate how model parameters influence the model outputs. Sensitivity analysis, in microalgae modelling has been used to reveal the impact of a parameter change on the model outputs [[2], [50], [51], [52]]. Dynamic sensitivity analysis in particular, can also help to determine which parts of the model are more significant ([53]; del [54]) and can allow the implementation of criteria to eliminate parameters [52,2]. Here we have used the L2 norm as our criterion of choice as explained in Section 3.3.

**Fig. 5:**
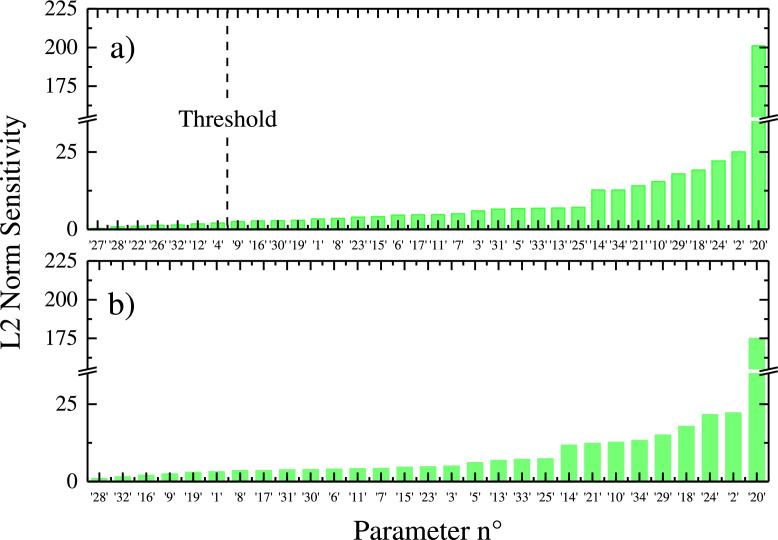
L2 Norm sensitivity (green bars) for all the parameters in (a) Model 34-P and (b) Model 29-P.

In Fig 5a the L2 norm of each column of the sensitivity matrix corresponding to each of the 34 parameters is shown. All of the parameters numbered in Fig. (5) are reported in Table (1). Five parameters with L2 norm below 2.5 (see threshold in Fig. (5a)) were considered for elimination as they were assumed to be “less” sensitive to input changes, resulting in a 29-parameter model. The L2 norms of the remaining 29 parameters are depicted in Fig. (5b). It should however be pointed out that parameters 27 and 28 are the saturation constants for intracellular and extracellular nitrates critical volume variation (Eq. (18), Eq. (19)), respectively, and 26 is the affinity regulator for the critical volume variation (Eq. (17)). The estimate for parameter 26 is around 0.22, as indicated in Table (1), and at this value the intracellular nitrate concentration has a larger effect on the model outputs than the extracellular nitrate concentration. However, the elimination of all of the parameters related to the critical volume variation implies a constant value for the critical volume, and it negatively influences the fitting and prediction capabilities for the average cell volume. Hence, the affinity factor was set to zero, and the extracellular dependence eliminated (Eq. (19)), reducing the critical volume dependence on intracellular nitrates only. Parameter 22 is related to the inhibition of nitrates consumption due to high light levels. Its value in the 34-parameter model of 738.8 seems to be clearly beyond the range of incident light used in this work and it has a small influence on the model outputs taking in to account the corresponding sensitivity in Fig. 8a. Based on these considerations, the light inhibition part in Eq. (31) was eliminated. Parameter 12 is related to the intracellular nitrate single-cell growth inhibition, and it has a small influence on the model results. Hence, the inhibition part for the high-affinity intracellular nitrates growth (Eq. (7)) is not considered in the 29-P model. Finally, parameter 4 is related to the lysis functionality. Due to its low sensitivity it was set to 1, without seeing any significant variation on the results. Further parameter eliminations leads to substantial changes in the model outputs, and in some cases could imply structural model modifications, which were avoided in this work.

The model parameters were hence successfully reduced to 29, and parameter estimation was carried out again to update their values, which can also be seen in Table 1. The estimation of the new parameters was necessary as after the elimination of the parameters, the model outcomes in terms of fitting and predictive capabilities were slightly deviating from original results. The new estimation could not have been necessary if the model was fully insensitive to the parameters elimination. However, the choice of a threshold in terms of sensitivity analysis for the model parameters number reduction is not a trivial matter, and the new estimation was necessary to evaluate if the reduced model after sensitivity analysis was still sufficiently able to describe the system considered.

The results in Fig. 4b, 4c, and 4c for the 29-parameter model highlight the good fit of the reduced parameter model. The predicted profiles of the 29-P model, especially for the first phase of the cell number growth, are slightly better than those obtained with the 34-P model as can be seen in Fig. 7a–c. This is also reflected in the improved prediction performance for cell densities depicted in Fig. 8.

In Table 2 the parameters derived from the literature are presented involving the partitioning function (Eq. (22)). The model results show good fitting and prediction capabilities for the whole range of the environmental conditions considered, including cell number predictions which are consistent with both the growth and the lysis phase.

**Fig. 6:**
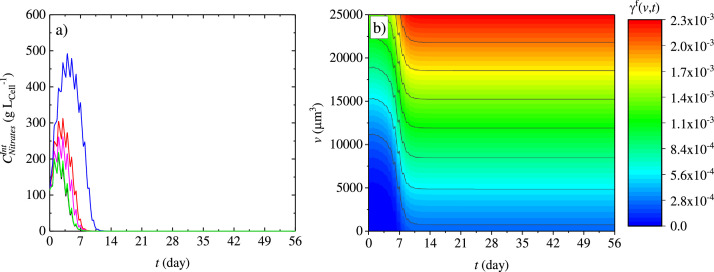
(a) Intracellular nitrates temporal profiles, black line N (–) L (BC), red line N (BC) L (BC), blue line N (+) L(BC), magenta line N(BC) L(++), and green line N(-) L (+)and (b) gamma function (Eq. (17)) variation with average cell volume and time.

Comparing the dynamics of cell number growth, with nitrates consumption it is evident that the growth does not stop as soon as the nitrates are depleted. Our model, by implementing Eq. (29), simulates the internal nitrates content, which is responsible for cell growth and depends mainly on the nitrates transported from the extracellular environment. As shown in Fig. 6a, higher internal nitrates content corresponds to higher external nitrates concentration. The consumption of the internal nitrates partially regulates, in the simulations, the duration of the growth process. The delay in the growth exhibited for the N (++) L (BC) case seems to be related to the inhibition of the nitrates consumption process, mainly controlled by the parameter $K_{{C_{NO_{3}^{-}}}^{Int},C:I}$ which has a value clearly in the range of the internal concentration for the high nitrates (N (++) L (BC)) case.

Interestingly, in both simulation and experiment with high light intensity (N (BC) L (++) growth seems to stop before the other cases, which in the simulation is related with higher consumption of intracellular nitrates, implemented by using a Monod function with saturation and inhibition kinetics (Eqs. (31) and (32)) for the average light intensity. $K_{I_{Ave},c:I}$ has a high value out of the range of the light intensities used in all of the experiments, indicating that probably light does not inhibit the intracellular nitrates consumption. Furthermore, as mentioned above, following sensitivity analysis the inhibition of the nitrate consumption due to the light intensity has been eliminated from the model in favour of a more straightforward Hill saturation function which works equally well.

The internal nitrogen concentration was not an *a priori* choice but was established based on modelling considerations. Microalgae can accumulate intracellular inorganic nitrogen [47,49] and also diatoms are able to do so [48]. This specific aspect allows microalgae to grow even when the nitrogen is depleted in the extracellular compartment. Consequently, this represents an important assumption when using kinetic models, because a direct proportionality to the depleted nutrient would cause the growth to stop earlier than it should. In light of this fact many researchers have utilised an expression derived from Droop [55] which considers an internal nutrient quota as the term responsible for growth, enabling the growth to continue even when the extracellular nitrogen content reaches zero. The droop model was successfully used both in structured and segregated models to describe the growth of microalgae in the case of nitrogen depletion ([[2], [50], [52], [56], [57], [58]]). However, the definition of nitrogen quota as the dynamic ratio between the nitrogen transferred in the intracellular compartment, and the biomass concentration implies that when the biomass content is decreasing due to cell death or lysis, the nitrogen quota increases. The latter does not have physical meaning if the nitrogen from the lysing cells will not go towards the remaining cells. Therefore, using the assumption that intrinsic nitrate concentration is conserved in the intracellular compartment, and considering this intrinsic concentration per unit volume, allows us to give a more appropriate physical meaning to the relation between nitrate content and cellular growth. As we can see in Fig. 6a, the intracellular nitrate contents drop to zero quickly after the rapid transport of nitrates ceases, and the use of double affinity kinetics (Eq. (6)) allows the growth to continue even in the low internal nitrates regime. Nevertheless, considering the intracellular nitrates concentration creates the need to perform dynamic intracellular concentration measurements, which further underpins the relevance of this work. Fig. 6b, depicts the gamma function (Eq. (15)) variation with time. When the intracellular concentrations are higher, the probability of having daughters is higher for larger cell volumes, and when the intracellular concentrations decrease, the probability becomes higher at lower cell volume values.

The above result is in line with the consideration that the fission critical volume increases in the presence of rich media composition as suggested in previous literature works for yeast [45,46].

### 4.3 Model predictive capabilities

**Fig. 7:**
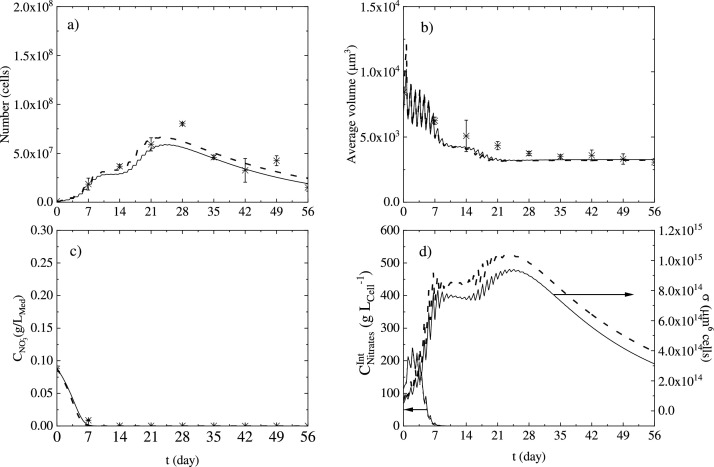
Experimental data against model predictions (Model P34 solid line, Model P29 dashed-line) for the experimental conditions N (-) L (+) for (a) cell number, (b) average volume, (c) extracellular nitrates concentration and (d) simulation results for intracellular nitrates profile and variance of cell density distribution.

**Fig. 8:**
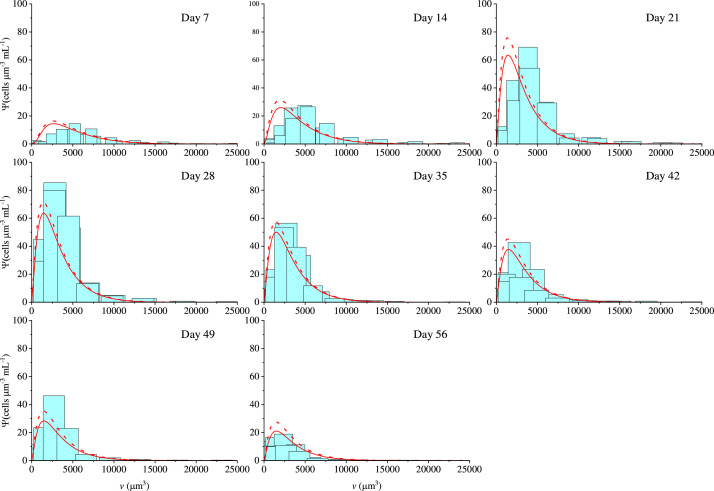
Predicted density distributions for model 34P (red lines) and 29P (dashed red lines) against experimental distributions (light blue bars) for 7, 14, 21, 28, 35, 42, 49, 56 days of cultivation.

The prediction capabilities of the model are tested against an independent experiment with different nitrates concentration and light intensity (N (-) L (+)), and the results are shown in Fig. 7a–c. Furthermore, a comparison between the experimental and the model cell density distributions is given in Fig. 8. To the best of our knowledge, microalgae cultivation modelling using population balances has only been used in a few research works. The majority of these consider single-cell growth in terms of mass and mass/age [59,56,60,29], while this work considers microalgae cultivation by using a volume-structured PBE. The cell number and the dry cell weight show two different patterns in batch cultivation systems. The cell number tends to decrease during nutrient depletion, and the dry cell weight continuously increases even after starvation occurs [61], which is also confirmed by our experiments (data not shown). The use of mass as a variable to descibe cell fission could lead to the wrong conclusion that cells are growing even in terms of cell number to increase the biomass content. The use of volume-based PBEs seems to be more appropriate from this point of view. Hence, as we can see in Fig. 7a, the cell number increases up to the point where there are enough intracellular nitrates to drive the growth process. When nitrates concentration goes towards depletion, both single-cell volume growth and fission phenomena slow down, until they stop due to complete nitrates depletion. These results are reflected in the kinetic expression in Eq. (8), which is included in the single-cell volume growth rate (Eq. (5)) and in the fission transition rate (Eq. (13)). The cell number achieves a peak after which the lysis period starts between 21 and 28 days. From a modelling point of view, the lysis phenomena are due to the kinetic term in Eq. (26), where the fission rate includes a negative exponential term, meaning that the lower the fission rate, the higher the lysis term will be. This explains the concept that when the cells face an environment where the multiplication is difficult, in this case, nutrient depletion, they are more likely to lyse. As we can see in Fig. 7a, the model results for the lysis phase, are in good agreement with the experimental data, leading to good prediction results for long cultivation times. As far as we know, lysis model-experimental data comparison results have not been reported in previous works on PBE modelling of microalgae cultivation [59,62,60,29]. Also, Fig. 7b reveals that the average cell volume decreases through the cultivation time and simulation data agree well with experiental results. Model predictions work well both in the initial and final phases of the cultivation stage, emphasizing the concept that cells modify their size depending on the environmental conditions they are exposed to.

The average cell volume decreases as the critical volume, $v_{c}$, decreases through the cultivation time (Eq. (17)), and the outcomes of this consideration are in agreement with previous research. Still, changes in the critical volume are related to the environmental conditions, and they cannot be reliably represented as a linear function of the average cell mass [62]. So, this work aims to give an explanation of the phenomena involved in the change of the critical cell volume. As it can be seen, the extracellular nitrate prediction in Fig. 7c agrees very well with experimental data. As depicted in Fig. 7d, the variance of the cell density distribution tends to increase during the growth phases, and to decrease during the lysis/stationary phases, indicating how the cells concentrate around a specific value of the cell volume. It can be also observed that during the first phase of the growth (growth phase in which internal nitrates concentration is higher, day 0–10) the variance grows quicker than during the second phase of the growth (phase in which the internal nitrates concentration is lower, day 10–25).

On the other hand, observing both Fig. 7b and d, the decrease in the variance in Fig. 7d corresponds to the phase where the average volume of the cells reaches a stationary value (Fig. 7b) indicating how the cells during the lysis phase tend to concentrate around the stationary value of the average volume. Fig. 8 depicts the model predictions in terms of density distributions against experimental data. The figure shows a good agreement in terms of number density. As can be seen in Fig. 7b, the average volume deviates more from experimental data for samples at day 14, 21, and 28. The cell lysis phase starting from day 35 onwards exhibits excellent agreement between experimental density distributions and model predictions. The calculation of the experimental cell densities is explained in the final part of Appendix B, Eq. B3 and B4. Overall, results including density distributions in terms of 0^th^ and 1^st^ order moments, and extracellular and intracellular nutrients concentrations, were presented in this section, which show a general good agreement with experimental results. The model demonstrates the ability to predict different phases of the microalgae cultivation, specifically the growth and lysis, which are both crucial parts of the cultivation process. Hence the model is not only useful for predicting microalgal growth at the cell population level, but can also be readily expanded to incorporate the prediction of microalgae metabolite concentrations.

## 5 Conclusions

A novel segregated-structured multi-parameter model was developed in this work. The objective was to describe growth and lysis phenomena during photoautotrophic growth of *H. pluvialis*, with particular emphasis on the phases where added-value compounds are produced. The model proposed a link between volume-based PBE and volume-based structure, also considering the influence of nutrient depletion on the microalgae growth and lysis. Model predictions for different cultivation conditions showed a good agreement with experiments for cell number and average cell volume. Moreover, predictions of the density distribution functions dynamics were shown, demonstrating the potential of the model for even microscopic scale applications. The inclusion of metabolites in the model, whose accumulation is inextricably linked to cell volume changes, is the next natural step in the development of this kind of structured-segregated models, and a more exhaustive analysis of intracellular concentrations could help to enforce the structured material balance concept. The model is aimed as a new tool for photoautotrophic cultivation design, and provides useful information for future implementation of optimisation of the cultivation processes for the bioproduction of targeted compounds. The efficient scale-up of microalgal cultivation systems is a particularly useful objective for future enhancements of the current modelling framework, which should also include considerations of the hydrodynamics of the cultivation apparatus, as well as light distribution efficiency, which is relevant for large-scale cultivation equipment.

## Declaration of Competing Interest

The authors declare that they have no known competing financial interests or personal relationships that could have appeared to influence the work reported in this paper.

## Data Availability

Data will be made available on request.

## Appendix Supplementary materials

Image, application 1
