# Supplementary material for: A multiscale modelling approach for Haematococcus pluvialis cultivation under different environmental conditions
Source: Biotechnol Rep (Amst). 2022 Oct 19;36:e00771. doi: 10.1016/j.btre.2022.e00771 (PMC9636539; doi:10.1016/j.btre.2022.e00771)
Supplement: Supplementary file 1 [file mmc1.docx]

# **Supplementary information**

## Appendix A. Numerical methods

Eq. (7) is a Partial Differential Integral Equation with variables $t$, and $v$. The equation is solved by using the method of lines in a Matlab environment. The internal variable $v$, is discretized within its domain between $v_{min}=0 {\mu m}^{3}$ and $v_{max}=25000 {\mu m}^{3}$. The discretization step $\Delta v$ is considered as constant, and it is calculated as in Eq. (A1), where $N_{V}$ is the total number of discretization points set to 100, which makes the simulations discretisation independent.

|  | $\Delta v=\frac{v_{max}-v_{min}}{N_{V}-1}$ | (A1) |
| --- | --- | --- |

The vector of the cell volume is then a function of $\Delta v$, and of the i^th^ domain point considered as follows:

|  | $v_{k}=\Delta v\cdot(k-1) k\in[1,N_{V}]$ | (A2) |
| --- | --- | --- |

Eq. (A3) is the discretisation of the main PBE presented in Eq. (1), including the most critical parts of the discretization. The derivative of the volume growth rate (Eq. (4)) is descritised using backwards finite differences following an up-wind scheme, and the integral of the birth term is solved using the trapezoid rule ensuring accuracy of the results with small computational effort. The system after using the method of lines results in a set of ODEs as follows:

|  | $\frac{d{\Psi_{V}}_{k}}{dt}=-\left( \frac{{r_{v}}_{k}{\Psi_{v}}_{k}-{r_{v}}_{k-1}{\Psi_{v}}_{k-1}}{\Delta v} \right)+\sum_{i=2,4}^{N} \theta_{i}i\left( \frac{1}{2}{\Psi_{V}}_{k}{\Gamma^{f}}_{k} {P_{i}}_{k,k}+\sum_{j=i+1}^{N_{V}-1} {\Psi_{V}}_{j}{\Gamma^{f}}_{j} {P_{i}}_{k,j}+\frac{1}{2}{\Psi_{V}}_{N_{V}}{\Gamma^{f}}_{N_{V}} {P_{i}}_{k,N_{V}} \right)- {\Psi_{V}}_{k}{\Gamma^{f}}_{k}-D_{k}{\Psi_{V}}_{k}$ | (A3) |
| --- | --- | --- |

The above is valid for $\forall i\in[2,N_{V}]$, and with ${\Psi_{V}}_{1}=0$. The corresponding initial condition is shown in Eq. (A4), $N^{0}$ being the total initial cell number.

|  | ${{\Psi_{V}}_{k}}^{0}=\frac{N^{0}}{V_{R}^{T}}\frac{1}{\sigma^{0}\sqrt{2\pi}}e^{\frac{-{(v_{k}-\mu^{0})}^{2}}{2{\sigma^{0}}^{2}}} k\in[1,N_{V}]$ | (A4) |
| --- | --- | --- |

The material balance in the intracellular and extracellular compartments includes a derivative term of the total cell volume as shown in Eq. (30) and Eq. (31). The derivative of the total cell volume in Eq. (30) is calculated by applying the Leibniz integration rule.

## Appendix B. Model outputs and experimental data

The population balance in Eq. (1) describes the evolution of the cell density $\Psi_{V}(v,t)$ through time. The fitting of the model against the experimental data is carried out by comparing cell the number and average volume as well as the extracellular nitrate concentration. The number of cells (referred to as the 0^th^ order moment of the cell density distribution function) is calculated as shown in the following equation:

|  | $cell number=V_{R}^{T}\cdot\int_{0}^{\infty} \Psi_{V}(v,t)\cdot dV$ | (B1) |
| --- | --- | --- |

Moreover, the ratio between the 1^st^ and the 0^th^ order moment of the cell desnity distribution is the average cell volume (cf. Eq. 32).

|  | $average cell volume=\frac{\int_{0}^{\infty} v\Psi_{v}dV}{\int_{0}^{\infty} \Psi_{V}dV}$ | (B2) |
| --- | --- | --- |

The software provided with the Nexcelom Cellometer Auto T4 cell counter (Nexcelom Bioscience) gives a histogram of the cell number $N_{i}^{exp}$regularly distributed ($\Delta d= constant$) in the diameter domain, with each class identified as $d_{i}$. Class $d_{1}$ is the first where cells are detected, and the last class $d_{Nclasses}$ is the last one where cells are detected. The nature of the model outputs implies the transformation of experimental data from the cell number histogram which is a function of cell diameters to a cell density which is a function of the cell volume. The first step is to add the zero class ($d_{0}=0$), and the last diameter class with cells in the histogram as $d_{Nclasses+1}=d_{Nclasses}+\Delta d$. Then the diameter domain is converted in a volume domain ($v_{i}=\left( 4/3 \right)\cdot\pi\cdot{({d_{i}}/2)}^{3}$). The new histogram is now depicts cell number $N_{i}^{exp}$distributed in volume classes $v_{i}$. The classes are not anymore regularly distributed due to the transformation from diameter to volume. The experimental cell density is defined as in Eq. (B3)

|  | ${{\Psi_{V}}^{Exp}}_{nc}=\frac{N_{nc}^{exp}}{\Delta v_{nc}V_{R}^{T}}$ | (B3) |
| --- | --- | --- |

$\Delta v_{nc}$ is defined as follows for the points of the new domain.

|  | $\Delta v_{nc}=\left[ v_{nc}+\left( \frac{v_{nc+1}-v_{nc}}{2} \right) \right]-\left[ v_{nc}-\left( \frac{v_{nc}-v_{nc-1}}{2} \right) \right] nc\in[1, N_{classes}]$ | (B4) |
| --- | --- | --- |
